# Optimizing growth and biomass production of non-*Saccharomyces* wine yeast starters by overcoming sucrose consumption deficiency

**DOI:** 10.3389/fmicb.2023.1209940

**Published:** 2023-06-06

**Authors:** Max Torrellas, Rocchina Pietrafesa, Aroa Ferrer-Pinós, Angela Capece, Emilia Matallana, Agustín Aranda

**Affiliations:** ^1^Institute for Integrative Systems Biology (I2SysBio), Universitat de València-CSIC, Paterna, Valencia, Spain; ^2^School of Agricultural, Forestry, Food and Environmental Sciences, University of Basilicata, Potenza, Italy

**Keywords:** non-*Saccharomyces* yeasts, sucrose, invertase, hydrolysis, bioreactor, wine fermentation

## Abstract

The use of non-*Saccharomyces* yeasts as starters in winemaking has increased exponentially in the last years. For instance, non-conventional yeasts have proven useful for the improvement of the organoleptic profile and biocontrol. Active dry yeast starter production has been optimized for *Saccharomyces cerevisiae*, which may entail problems for the propagation of non-*Saccharomyces* yeasts. This work shows that the poor growth of *Hanseniaspora vineae* and *Metschnikowia pulcherrima* in molasses is related to a deficient sucrose consumption, linked to their low invertase activity. In order to address this issue, simple modifications to the cultivation media based hydrolysis and the reduction of sucrose concentration were performed. We performed biomass propagation simulations at a bench-top and bioreactor scale. The results show that cultivation in a hexose-based media improved biomass production in both species, as it solves their low invertase activity. The reduction in sugar concentration promoted a metabolic shift to a respiratory metabolism, which allowed a higher biomass yield, but did not improve total biomass production, due to the lower sugar availability. To evaluate the technological performance of these adaptations, we performed mixed grape juice fermentations with biomass produced in such conditions of *M. pulcherrima* and *S. cerevisiae.* The analysis of wines produced revealed that the different treatments we have tested did not have any negative impact on wine quality, further proving their applicability at an industrial level for the improvement of biomass production.

## 1. Introduction

During wine fermentation, yeast metabolism is necessary for the transformation of sugars into ethanol and CO_2_ through alcoholic fermentation. Besides from alcohol, during fermentation yeasts species produce diverse secondary volatile metabolites that are involved in wine flavor (Capece and Romano, 2019). The most common practice in wineries around the world is the inoculation of grape musts with pure starter cultures of *Saccharomyces cerevisiae* in the form of active dry yeast, which ensure a complete sugar fermentation and produce wines with a consistent and reproducible quality between seasons (Pérez-Torrado et al., 2015). In recent years, there has been an increasing demand of more complex and characteristic wines by consumers and producers alike, which has increased the interest in non-*Saccharomyces* wine yeasts due to their effect on wine volatile composition (Lambrechts and Pretorius, 2000; Ciani et al., 2010; Comitini et al., 2011). Many authors have explored the use of many different non-*Saccharomyces* species in wine fermentation, centered around three main goals, the production of wines with enhanced organoleptic profiles, the reduction in ethanol content and the biocontrol of spoilage microorganisms (Jolly et al., 2014; Varela, 2016; Morata, 2019). In fact, research has led to the development of many commercially available non-*Saccharomyces* starter cultures (Roudil et al., 2020). Nonetheless, most non-*Saccharomyces* yeasts have weak fermentative power (Fleet, 2008) and they are restricted to being used alongside *S. cerevisiae* in mixed or sequential fermentations for a complete fermentation of grape must sugars (Varela, 2016). Among non-*Saccharomyces* yeasts, *Hanseniaspora vineae* is interesting within its genus due to its high fermentative capacity (Martin et al., 2018). Another point of interest is its β-glucosidase and β-xylosidase activities, which allow the liberation of aromatic compounds from grape precursors (López et al., 2015; Hu et al., 2016). Wines produced in mixed fermentations with *H. vineae* have shown a higher accumulation of desired volatile compounds, such as 2-phenilethyl acetate, ethyl lactate or α-terpineol, which result in wines with higher scores in tasting panels (Viana et al., 2011; Lleixà et al., 2016). Another widely studied non-*Saccharomyces* species in wine production is *Metschnikowia pulcherrima*. This yeast species has a potential use as a biocontrol agent against spoilage yeasts (Oro et al., 2014; Morata et al., 2019). Wines produced in mixed fermentations with *M. pulcherrima* present a more complex aromatic profile than wines produced only with *S. cerevisiae* (Benito et al., 2015; Ruiz et al., 2018; Seguinot et al., 2020). *Metschnikowia pulcherrima* has also been successfully used for ethanol reduction in mixed fermentations (Contreras et al., 2014; Quirós et al., 2014; Puškaš et al., 2020). The controlled aeration of the must allow sugar respiration during the initial stages of fermentation by *M. pulcherrima*, although this technique may have a negative effect on wine sensory profile (Tronchoni et al., 2018).

Commercially available active dry yeast starters are produced at industrial level in a process that has been optimized for the production of *S. cerevisiae,* generally using beet or sugarcane molasses as a substrate (Pérez-Torrado et al., 2015). Molasses are a particularly interesting substrate due to their reduced cost and high sugar concentration (65%–75% sucrose; Pérez-Torrado et al., 2015), although they should be supplemented with a nitrogen source and vitamins for optimal growth (Pérez-Torrado et al., 2015). Furthermore, *S. cerevisiae* can efficiently hydrolyze sucrose and transport and metabolize the resulting monosaccharides, glucose and fructose. Sucrose is hydrolyzed by invertase, an enzyme coded by the *SUC* (*SUC1–SUC5* and *SUC7*) gene family (Carlson and Botstein, 1983), of which *SUC2* is the only one active in laboratory strains. *SUC2* expression is tightly regulated by catabolite repression. *SUC2* expression is repressed at high glucose concentrations (2%) and derepressed by the AMPK/SNF1 pathway when glucose concentrations drop (below 0.1%; Özcan et al., 1997). During biomass propagation yeasts suffer many different stresses that affect the viability and technological performance of active dry yeast (Matallana and Aranda, 2016). In particular, the dehydration step is notably damaging, and causes a reduction in cell viability and vitality (Dupont et al., 2014; Rapoport et al., 2019). *Saccharomyces cerevisiae* is well adapted to the industrial biomass propagation process. However, different non-*Saccharomyces* species show diminished growth and reduced tolerance to dehydration under laboratory scale simulations of the biomass propagation process when compared to *S. cerevisiae* (Torrellas et al., 2020). This is particularly the case for *H. vineae*, that shows very poor tolerance to dehydration, while *M. pulcherrima* shows high viability after drying. In fact, despite the widespread use of molasses as a substrate for industrial wine yeast production, it may not be the ideal propagation media for some non-*Saccharomyces* yeasts. It has been described that some species are unable to properly metabolize sucrose. In fact, commercially available strains of *M. pulcherrima* seem to be unable to consume sucrose (Lu et al., 2016; Chua et al., 2018) and complete growth in sucrose-rich mediums can only be achieved when pretreated with exogenous invertase (Schnierda et al., 2014). The same is true for *H. vineae*, which is unable to grow with sucrose as the only carbon source (Bellut et al., 2018). In this work, we aim to characterize the behavior of two non-*Saccharomyces* species in biomass propagation simulations in relation with sucrose consumption and metabolic adaptation. We have tested two simple modifications to the propagation process to try to improve biomass production and we have analyzed their effect on wine quality by performing mixed fermentations with the produced dried biomass.

## 2. Materials and methods

### 2.1. Strains and cultivation conditions

Two non-commercial non-*Saccharomyces* strains provided by Lallemand Inc. (Montreal, Canada) were analyzed: *Hanseniaspora vineae* and *Metschnikowia pulcherrima*. The commercial *Saccharomyces cerevisiae* strain Lalvin T73 (Querol et al., 1992) was used as the reference strain.

Precultures for biomass propagation were prepared in liquid YPD medium (1% (w/v) yeast extract, 2% (w/v) peptone, 2% (w/v) glucose) and incubated at 30°C with shaking (180 rpm). Growth curves were followed in a Varioskan plate reads (Thermo Scientific). Biomass propagation experiments were performed using beet molasses provided by Lessafre Iberica (60 g/L of sucrose for batch experiments, 100 g/L of sucrose for fed-batch phase and 20 g/L in diluted molasses experiments), supplemented with 7.5 g/L (NH_4_)_2_SO_4_, 3.5 g/L KH_2_PO_4_, 0.75 g/L MgSO_4_, and 10 mL/L vitamin solution (Torrellas et al., 2020). The vitamin solution contained 0.5 mg/L D-biotin, 1 mg/L calcium pantothenate and 1 mg/L thiamine hydrochloride. Molasses and mineral solutions were autoclaved separately at 121°C for 20 min. The vitamin solution was filter sterilized (0.2 μm pore size). pH was adjusted to 4.5 using H_3_PO_4_ 42.5% (v/v). To analyze sucrose acid hydrolysis, concentrations of HCl ranging from 0.5% to 2% (v/v) and incubation times of 15, 30, and 60 min and temperatures of 80°C and 95°C were tested (Supplementary Table 1). Molasses treated with HCl 1% (v/v) for 1 h at 80°C was enough to fully hydrolyze sucrose into glucose and fructose, so this condition was used in the next experiments. After treatment. pH was adjusted at 4.5 using NaOH 5 M. Diluted molasses were treated as above lowering sucrose concentration from 60 to 20 g/L.

For bench-top scale biomass propagation experiments, cells were cultivated in flasks at 30°C with shaking (180 rpm) in molasses and in liquid YPD medium. Bioreactor scale assays were performed using a 5 L ez2-Control bioreactor (Applikon Biotechnology, Netherlands) equipped with proportional, integral and derivative (PID) control units for pH, temperature, oxygen and agitation speed. The bioreactor containing 2 L of sterilized molasses was inoculated with an initial OD of 0.1 from YPD precultures. Antifoam 0.05 and (w/v; antifoam 204, Sigma) was added. Cells were cultivated at 30°C with stirring. Dissolved oxygen was measured with an electrode and maintained at 20% by a PID control system that allowed automatic modification of the air flux and the agitation speed between (300–500 rpm). The initial pH was 4.5 and it was allowed to freely vary during the batch phase. During the fed-batch phase pH was maintained at 4.5 by automatic addition of 1 M NaOH or 42.5% H_3_PO_4_. Cell growth was monitored by measuring OD_600_.

### 2.2. Biomass dehydration and rehydration conditions

Yeast biomass was separated from molasses by centrifugation at 4000 rpm and several washes with sterile distilled water were performed. The biomass paste was recovered and placed as thin noodles inside a tabletop fluid bed dryer (Sherwood Scientific, Cambridge, United Kingdom). Biomass was dehydrated with an air flux of 2.5 m^3^/min at 37°C during 50 min for *S. cerevisiae*, 40 min for *H. vineae* and 60 min for *M. pulcherrima,* the time needed to reach 8% humidity, as determined by weight loss. It was then stored at 4°C. For rehydration, the dry biomass was placed in sterile distilled water (1 part biomass, 9 parts water) at 37°C for 10 min, followed by 10 min with shaking at 140 rpm.

The biomass from the molasses culture (fresh cells) and the rehydrated biomass (dry cells) were diluted, plated on YPD plates and incubated for 24 h at 30°C, after that colony-forming units (CFU) were counted. The survival percentage was calculated by taking the CFU of the fresh cells as 100% survival.

### 2.3. Metabolite determination

Sucrose determination was performed by mixing 40 μL of each sample with 160 μL of a sodium acetate buffer 50 mM pH 5.0 containing invertase 2.5 U (Sigma, United States). Samples were then incubated at 30°C for 10 min. The reaction was stopped by adding 100 μL K_2_HPO_4_ 0.4 M and keeping samples at 95°C for 3 min. The glucose liberated by the reaction was determined by the glucose oxidase-peroxidase method. One hundred μL of each sample was added to 400 μL of the GOX/P reactive (glucose oxidase 7.8 U, peroxidase 0.4 U, o-dianisidine 0.9 mM in 100 mM pH 7.0 potassium phosphate buffer). Reaction was performed for 15 min at 30°C and was stopped by the addition of 500 μL of HCl 6 N. OD_540_ was measured and glucose content was calculated using a calibration curve (glucose 0–10 mg/mL). Reducing sugars were determined following the protocol described by Robyt and Whelan (1972). In short, 100 μL of DNS [3,5-dinitrosalicilic acid 1% (w/v), NaOH 1.6% (w/v), potassium-sodium tartrate 30% (w/v)] were added to 100 μL of each sample. Samples were incubated at 95°C for 5 min and cooled in an ice bath. one mL of water was added to each sample and OD_540_ was measured. Reducing sugars content was calculated using a calibration curve (glucose 0–2 g/L). Enzymatic ethanol determination was performed by the spectrophotometric detection at 340 nm of the NADH formed during ethanol oxidation to acetaldehyde by alcohol dehydrogenase. Two hundred μL of each sample were mixed with 800 μL of a buffer containing glycine 0.2 M, Tris 0.3 M pH 9.7, NAD^+^ 2 mM. Initial OD_340_ was measured and used as a blank for each sample. Yeast alcohol dehydrogenase 20 U/mL (Sigma, United States) was added and samples were incubated at room temperature for 15 min. OD_340_ was measured and ethanol concentration was determined using a calibration curve (ethanol 0–0.9 mM).

### 2.4. Invertase activity

Invertase activity was determined following the protocol described by Harkness and Arnason (2014). In short, cell cultures were prepared for initial exponential growth (OD_600_ 0.2–0.3) in YPD. Once cultures reached the desired OD, 1 × 10^6^ cells were collected to test their activity under invertase-repressing conditions. The remaining cells were washed and transferred (initial OD_600_ 0.2–0.3) to an YPD medium containing 0.05% (w/v) glucose or to molasses medium and incubated at 30°C for 2 h with shaking (250 rpm). 1×10^6^ cells were then collected to test activity under derepressing conditions. Cells were resuspended in 50 μL of sodium acetate 50 mM pH 5.1 and 12.5 μL of sucrose 0.5 M were added. Samples were incubated at 37°C for 10 min and the reaction was stopped by the addition of 75 μL of K_2_HPO_4_ 0.2 M. Samples were kept at 95°C for 3 min and then transferred to an ice bath. The liberated glucose was determined by the glucose oxidase-peroxidase method (see above).

### 2.5. Wine fermentations

*Metschnikowia pulcherrima* and *S. cerevisiae* were tested in mixed fermentations at laboratory scale using simultaneous inoculation. Fermentations were carried out in natural red grape must, Primitivo grape variety (sugar concentration 225 g/L, available nitrogen 228 mg N/L, pH 3.46). The grape must was pasteurized for 20 min at 90°C. Fermentations were carried out in 100 mL Erlenmeyer flasks containing 100 mL of must, giving around 35 mL of head space. In each fermentation, *S. cerevisiae* was co-inoculated with *M. pulcherrima* previously cultured under different conditions, by using a different inoculation ratio (10^3^ viable cells/mL for *S. cerevisiae* and 10^7^ viable cells/mL for *M. pulcherrima*). As a control, pure fermentations with *S. cerevisiae* (10^7^ viable cells/mL) were performed. All the experiments were carried out in triplicate at 28°C without shaking. The fermentation was monitored by evaluating CO_2_ evolution by weight loss and by evaluation of yeast viable populations. The fermentation process was considered completed when samples reached a constant weight. Fermenting must samples were taken from each flask at days 0, 1, 2, 4, 7, 8, 9, 10, 11, and 14 of fermentation. Each sample was diluted in saline solution [NaCl 0.85% (w/v)] and plated on Wallerstein Laboratory Nutrient Agar medium (WL, Pallmann et al., 2001), which allows yeast species differentiation by colony morphology and color. Conventional chemical parameters (sugars, total acidity, ethanol and pH) in must under fermentation and in final wines were determined by Fourier-transform Infrared (FTIR) spectroscopy, using an OenoFoss detector (Foss Analytics, Denmark). The concentration of main secondary compounds (acetaldehyde, n-propanol, isobutanol, n-propanol, n-butanol, isoamyl alcohol, acetoin, acetic acid and ethyl acetate) in wines was determined by gas chromatography, as previously described (Capece et al., 2013).

### 2.6. Statistical analysis

Each test was carried out independently in triplicate, and the results are represented as the average with the corresponding standard deviation (±SD). Analysis of variance (ANOVA) using Tukey’s test and Principal Component Analysis (PCA) was carried out with PAST software ver. 4.09. The results were considered significant at *p-*value ≤ 0.05.

## 3. Results

### 3.1. Slow sucrose consumption in non-*Saccharomyces* species is related to low invertase activity

**Figure 1:**
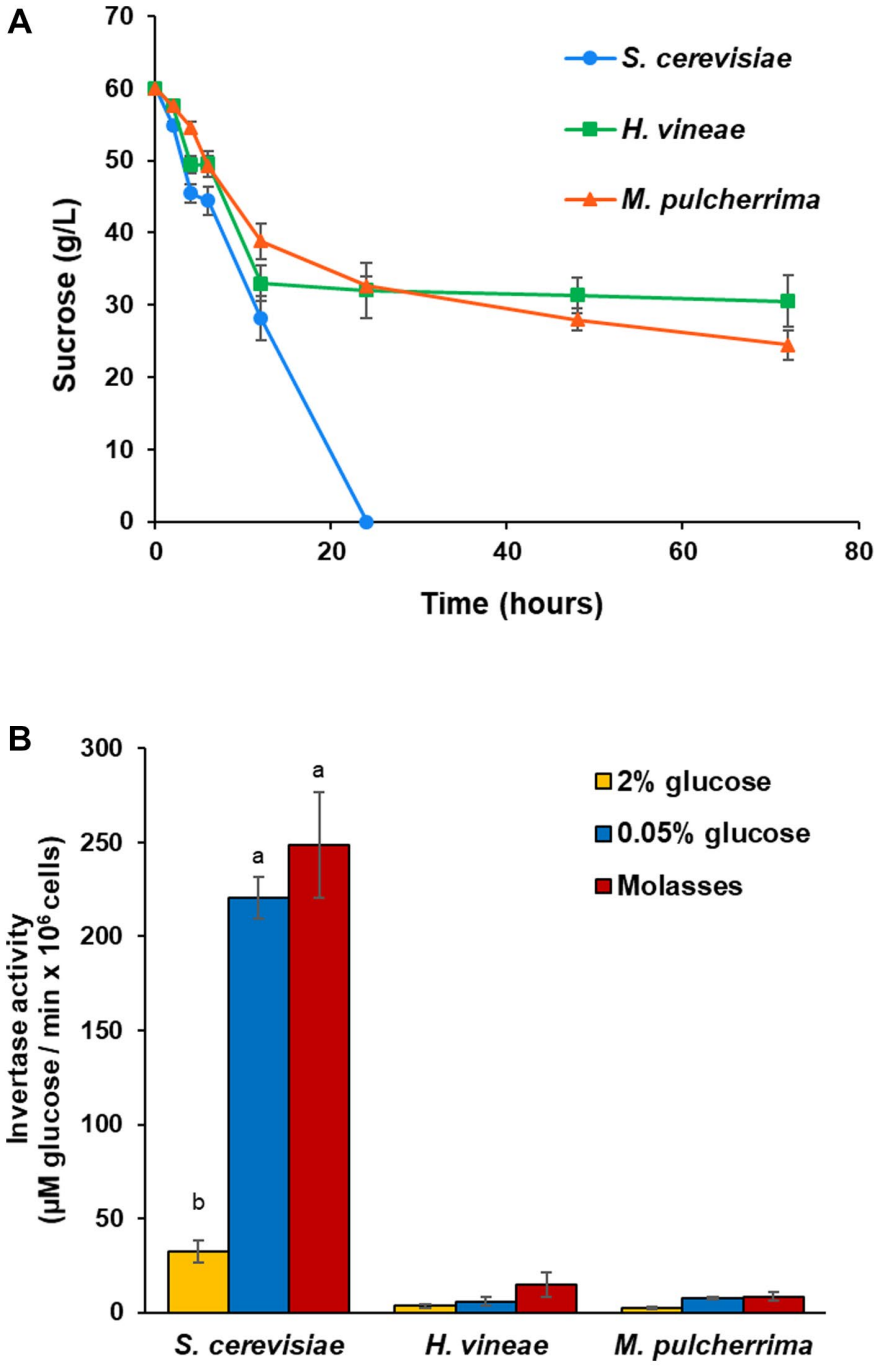
Sucrose consumption and invertase activity. **(A)** Sucrose levels (g/L) in molasses during growth trials in molasses. **(B)** Invertase activity, expressed as μM of glucose liberated by 10^6^ cells per minute. Invertase activity was measured under *SUC2* repressing conditions (2% glucose) and derepressing conditions (0.05% glucose and in molasses). Error bars correspond to the SD of three independent experiments. Different letters denote statistical differences among species and conditions (*p* < 0.05).

The aim of this study is understanding how media composition and growth conditions affect the technological behavior of wine yeasts during biomass propagation, as well as performing simple and easily adaptable modifications to the process to improve biomass yield. Based on the differences in growth between *H. vineae* and *M. pulcherrima* and a commercial *S. cerevisiae* strain described in previous work (Torrellas et al., 2020), we measured sucrose levels in molasses during biomass propagation simulations. As shown in Figure 1A, sucrose consumption in *S. cerevisiae* is rapid as it is depleted at around 24 h of cultivation. On the contrary, *H. vineae* and *M. pulcherrima* show an initial consumption rate similar to *S. cerevisiae,* but the consumption rate slows down after around 20 h and levels remain high (around 30 g/L of the original 60 g/L) even after long cultivation times.

In order to elucidate if the cause of the different sucrose consumption profiles was due to a defect in the ability to metabolize sucrose, we studied invertase activity in repressing (2% glucose) and derepressing (0.05% glucose) conditions (Figure 1B). Invertase activity differences between *S. cerevisiae* and non-*Saccharomyces* yeasts are evident. Enzymatic activity was lower in non-*Saccharomyces* species than in *S. cerevisiae* in both conditions. All three species showed a statistically significant increase in activity under derepressing conditions, with a 7-fold induction for *S. cerevisiae*, and a 4-fold induction in *H. vineae* and *M. pulcherrima.* Even then, enzymatic activity for both non-*Saccharomyces* species was much lower than in *S. cerevisiae* under repressing conditions. This lack of an adequate invertase activity helps to explain their different behavior in laboratory scale biomass propagation simulations. The activity was measured also in molasses that are supposed to be non-repressing conditions. It does indeed induce invertase activity in all species, particularly in *H. vineae*.

**Figure 2:**
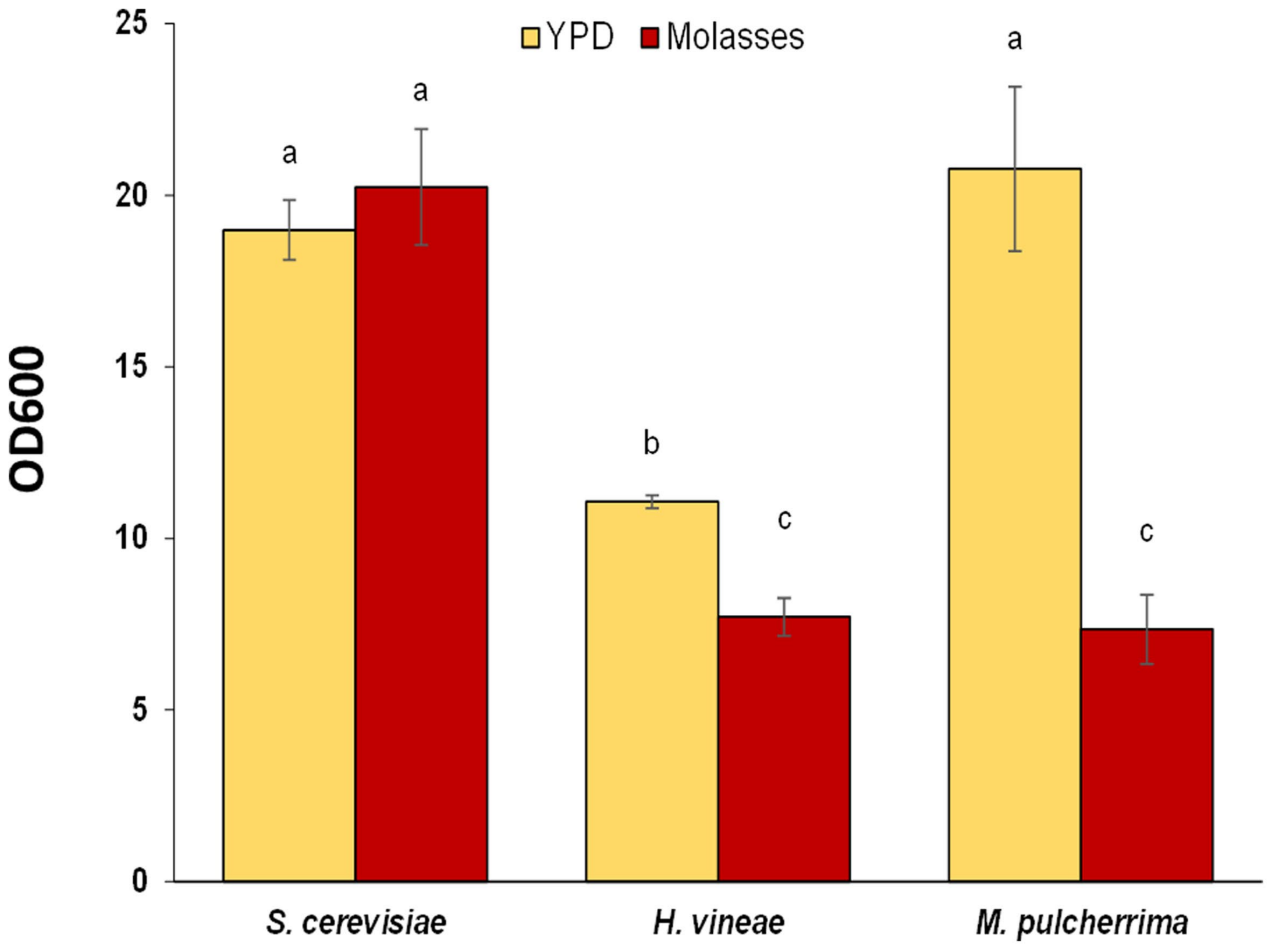
Cellular growth (OD_600_) after 24 h in liquid YPD medium and in molasses. Error bars correspond to the SD of three independent experiments. Different letters denote statistical differences among species and conditions (*p* < 0.05).

As a first approach to understand and improve biomass production of the non-*Saccharomyces* species, we tested the effect of a different cultivation media on cellular growth by using YPD, a standard laboratory media (Figure 2). The change in media has a positive effect on the cellular growth of both non-*Saccharomyces* species, while no differences were detected for *S. cerevisiae*. *H. vineae* reached OD values that were 1.4 fold higher than in molasses, while in *M. pulcherrima* the increase was more pronounced (2.8 fold). In fact, OD values in *M. pulcherrima* cultured in YPD were statistically similar to those of *S. cerevisiae* in both media. The main carbon source in YPD is glucose, at a lower concentration to that of sucrose in molasses (20 g/L vs. 60 g/L). The increase in cellular growth in both non-*Saccharomyces* is likely a consequence of a more efficient glucose metabolism, which can be imported to the cellular interior and metabolized efficiently.

**Figure 3:**
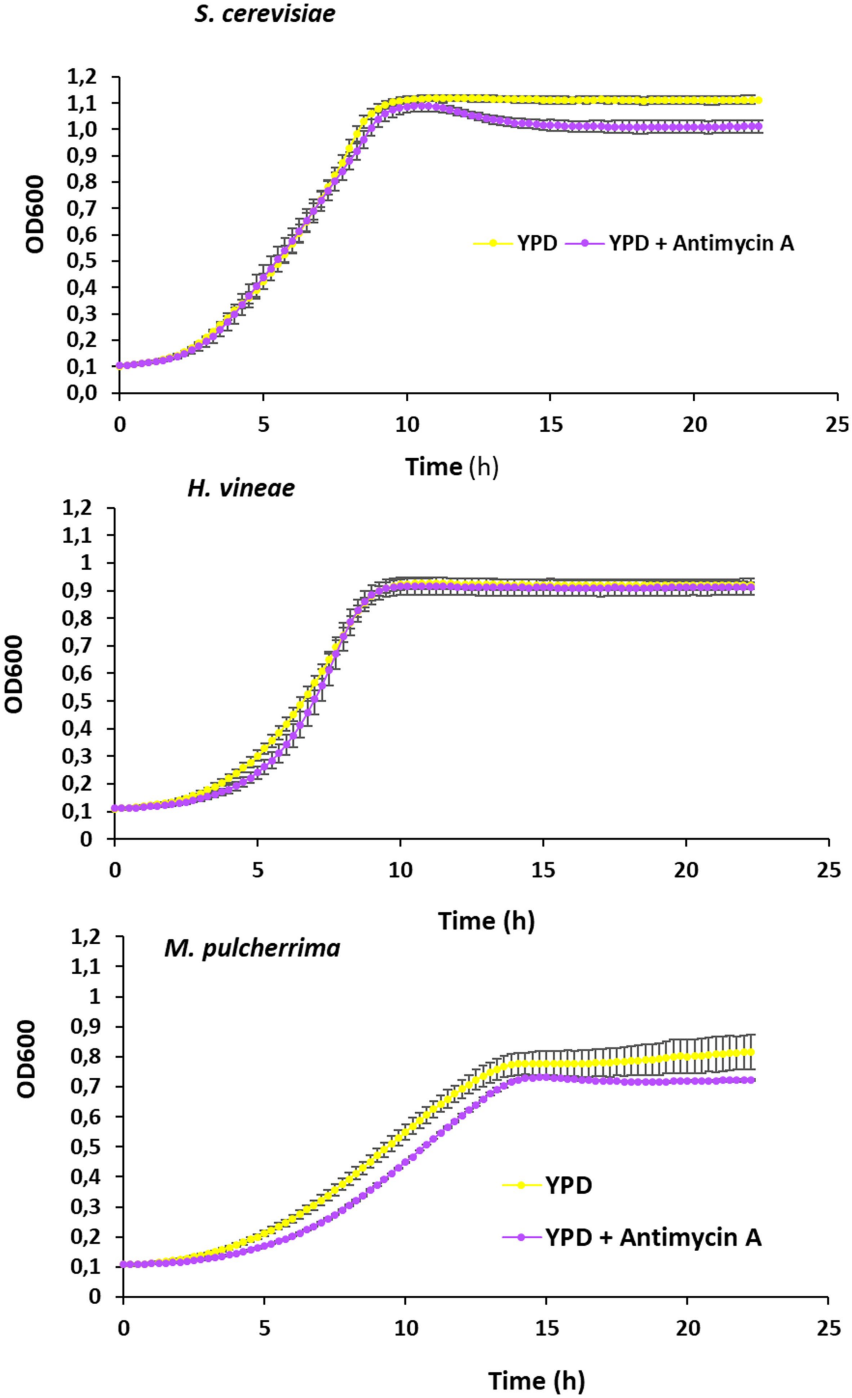
Analysis of respiratory metabolism. Growth curves in YPD and YPD + antimycin A (3 μg/m) of *Saccharomyces cerevisiae*, *Hanseniaspora vineae*, and *Metschnikowia pulcherrima*. Experiments were carried out in triplicates and mean and standard deviation is shown.

Growth in the standard laboratory medium allows us a better understanding of the physiology of the yeasts of interest. The addition of the inhibitor of the cytochrome C reductase antimycin A block the electron transport chain, providing information on the role of mitochondrial respiration in those yeasts. Growth curves in microwell plates were obtained (Figure 3). In *S. cerevisiae* during exponential growth there is no impact of antimycin A. That confirms that cells are fermenting glucose as main source of energy. However, antimycin A impact the entry in stationary phase, and at longer times, when the diauxic shift is expected and metabolism turns to respiration, a decrease in optical density is observed. *H. vineae* shows a consistently fermentative behavior, and there are no big differences throughout all the growth curve. Addition of antimycin A to *M. pulcherrima* leads to both a delay in exponential growth and to a lower maximal cell density, so this species has a more respiratory metabolism that the other two.

### 3.2. Simple modifications to the biomass propagation media improve biomass yield and viability of *Hanseniaspora vineae* and *Metschnikowia pulcherrima*

Sucrose being the main carbon source in molasses seems to be the main hindrance for a proper growth of wine yeasts that are unable to efficiently catabolize this sugar. Despite the improvement in biomass production described previously, standard rich media often used in the laboratory, such as YPD, cannot be used at an industrial scale due to their high economic cost. In order to address both of these issues, we tested two treatments that aimed to improve biomass production in non-*Saccharomyces* species while keeping molasses as the basis of the growth media.

**Figure 4:**
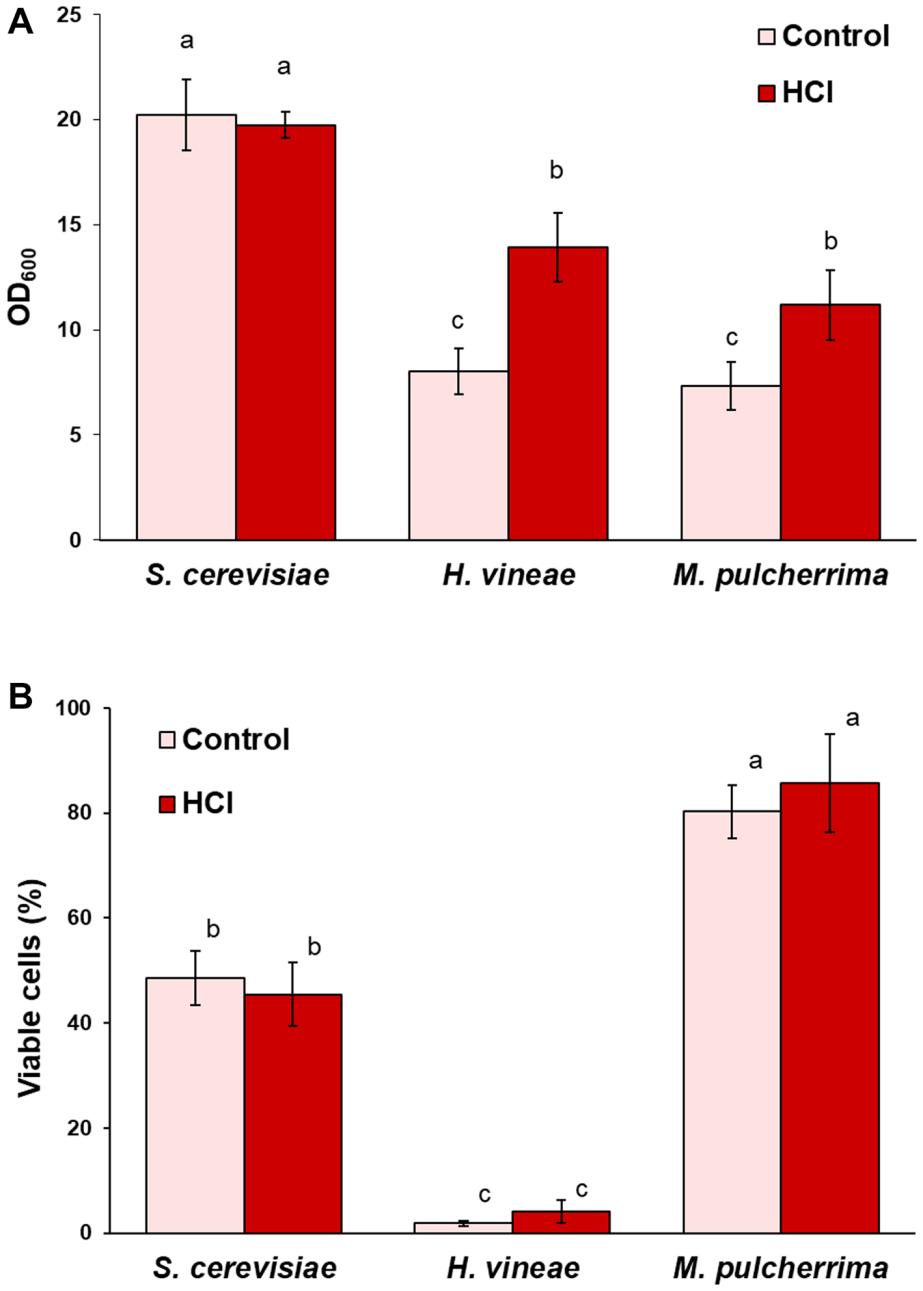
Effect of cultivation in hydrolyzed molasses. **(A)** Cell growth (OD_600_) in control and hydrolyzed molasses after 24 h at 30°C with shaking. **(B)** Viable cells after dehydration. Error bars correspond to the SD of three independent experiments. Different letters denote statistical differences between species and conditions (*p* < 0.05).

In first place, we tested acid hydrolysis. Growth was followed by measuring OD_600_ after 24 h, when *S. cerevisiae* reaches saturation (Torrellas et al., 2020). As shown in Figure 4A, the change in carbon source had a positive effect on cellular growth on both non-*Saccharomyces* species, while it had no effect on *S. cerevisiae*, due to its optimal sucrose consumption. Sucrose hydrolysis seems to solve the issue of low invertase activity, at least in the case of *H. vineae*, in which, reducing sugars were completely depleted after 24 h of cultivation, as it occurs in *S. cerevisiae* (data not shown). On the other hand, sugar consumption in *M. pulcherrima* was incomplete at 24 h of cultivation, with a remaining concentration of 45.67 (± 2.77) g/L of reducing sugars out of the initial 61.35 (± 11.20) g/L, similar to what was observed for sucrose consumption (Figure 1A). In this species, other factors aside from low invertase activity may be at play in determining the sugar consumption rate, such as a differential metabolic regulation that may cause a slower sugar uptake and slower growth. Furthermore, hydrolyzed molasses did not have any negative effect on biomass yield in any of the three species (Table 1). Due to the relevance of dehydration in determining biomass usability in industry, we tested the effect of molasses hydrolysis on cell viability after dehydration. The treatment with HCl did not have any negative effect on cell viability in any of the three species (Figure 4B), being *H. vineae* very sensitive to this process as previously described (Torrellas et al., 2020).

**Table 1:** Biomass propagation parameters under different conditions.

Species	Condition		Biomass yield (g biomass/g sucrose)	Max. ethanol production (g/L)	Viable cells after dehydration (%)
*Saccharomyces cerevisiae*	Molasses	Bench-top	0.09 (± 0.01)^e^	27.72 (± 2.39)^a^	48.55 (± 5.11)^c^
		Bioreactor (batch)	0.08 (± 0.01)^e^	23.75 (± 2.40)^a^	40.17 (± 4.74)^c^
		Bioreactor (fed-batch)	0.14 (± 0.02)^d^	n.d.	66.76 (± 8.23)^b^
	Molasses + HCl	Bench-top	0.08 (± 0.01)^e^	25.78 (± 1.16)^a^	45.39 (± 6.06)^c^
	Diluted molasses (20 g/L)	Bench-top	0.09 (± 0.01)^e^	10.54 (± 0.35)^c^	43.18 (± 4.89)^c^
*Hanseniaspora vineae*	Molasses	Bench-top	0.06 (± 0.01)^e^	7.15 (± 1.47)^d^	1.83 (± 0.51)^d^
		Bioreactor (batch)	0.08 (± 0.02)^e^	n.d.	43.25 (± 10.99)^c^
	Molasses + HCl	Bench-top	0.06 (± 0.01)^e^	16.07 (± 2.39)^b^	4.16 (± 2.19)^d^
		Bioreactor (batch)	0.09 (± 0.02)^e^	18.54 (± 1.22)^b^	36.81 (± 1.33)^d^
	Diluted molasses (20 g/L)	Bench-top	0.21 (± 0.01)^c^	0.23 (± 0.80)^f^	44.27 (± 7.27)^c^
		Bioreactor (batch)	0.57 (± 0.08)^a^	n.d.	53.54 (± 8.52)^c^
*Metschnikowia pulcherrima*	Molasses	Bench-top	0.05 (± 0.01)^e^	3.12 (± 0.46)^e^	80.29 (± 5.07)^a^
		Bioreactor (batch)	0.33 (± 0.04)^b^	n.d.	85.85 (± 2.94)^a^
	Molasses + HCl	Bench-top	0.07 (± 0.02)^e^	1.68 (± 0.91)^f^	85.65 (± 9.36)^a^
		Bioreactor (batch)	0.23 (± 0.04)^c^	n.d.	90.11 (± 5.36)^a^
	Diluted molasses (20 g/L)	Bench-top	0.34 (± 0.02)^b^	2.17 (± 0.19)^f^	89.32 (± 1.98)^a^
		Bioreactor (batch)	0.41 (± 0.05)^b^	n.d.	92.27 (± 7.86)^a^

Biomass yield (g biomass/g sucrose), maximum ethanol production (g/L), and cell viability after dehydration (%) were measured in bench-top and bioreactor growth tests using different growth media: molasses (control conditions), hydrolyzed molasses (molasses + HCl), and diluted molasses. All the measurements were taken in triplicate. Different letters denote a significant difference among species and conditions within the same parameter (*p* < 0.05). N.d., not detected.

**Figure 5:**
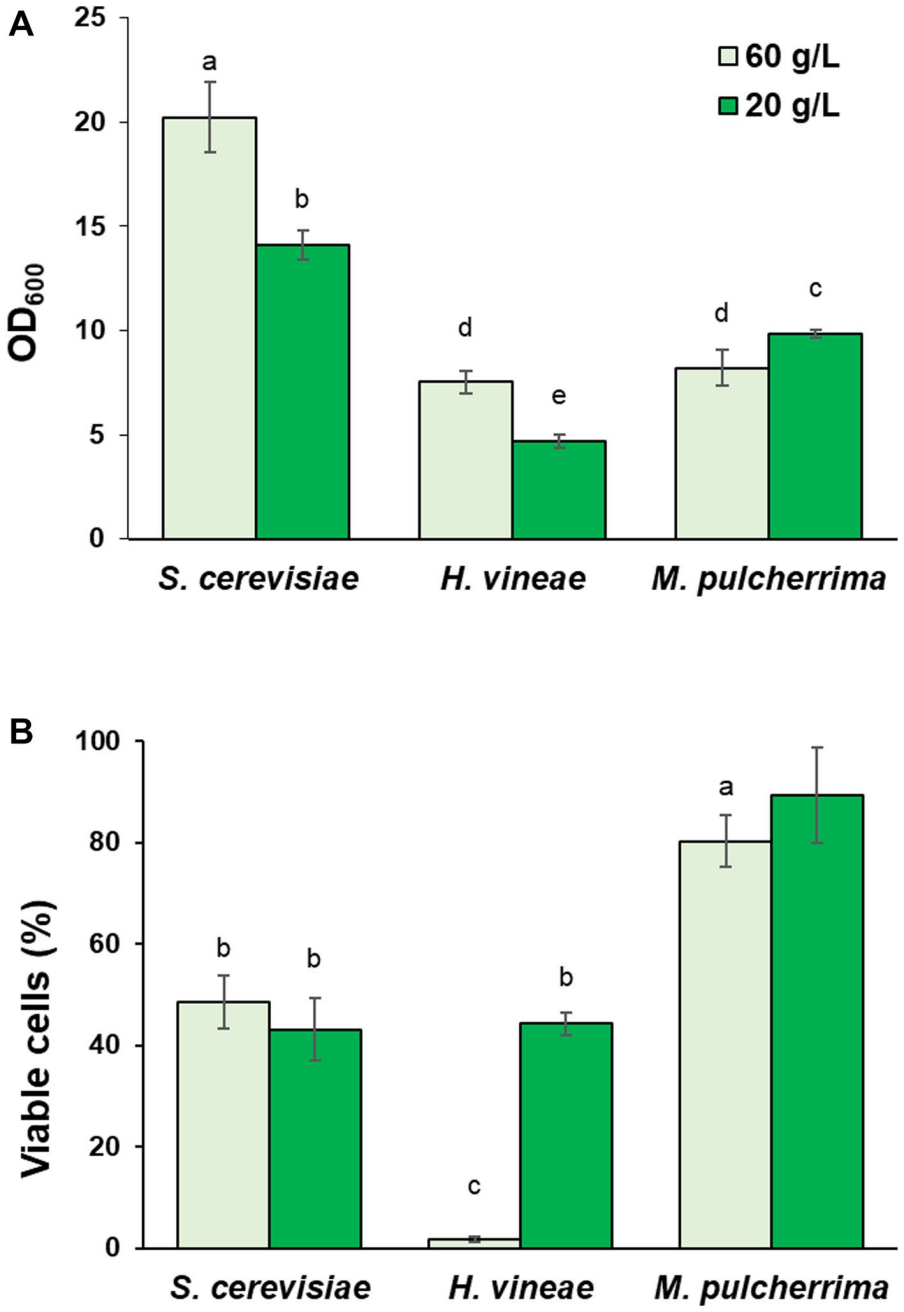
Effect of cultivation in molasses diluted to 20 g/L of sucrose. **(A)** Cell growth (OD_600_) in control (60 g/L) and diluted (20 g/L) molasses after 24 h at 30°C with shaking. **(B)** Viable cells after dehydration. Error bars correspond to the SD of three independent experiments. Different letters denote statistical differences between species and conditions (*p* < 0.05).

As an alternative to molasses hydrolysis, in order to test the possible effect on metabolic regulation due to carbon source levels, we studied the effect of the reduction in sucrose concentration. As shown in Figure 5A, *S. cerevisiae* and *H. vineae* showed diminished growth when cultivated with a lower sucrose concentration, which could be expected due to the limitation in carbon source, although growth reduction was not proportional to the reduction in sucrose concentration, suggesting an increase in biomass due to a different metabolism, probably involving a higher rate of respiration. Interestingly, *M. pulcherrima* showed increased growth under these conditions. Aside from the effect on cellular growth, sucrose dilution had a significantly positive effect on cell viability after dehydration in *H. vineae* (Figure 5B). Viability in diluted molasses reached levels similar to those of the commercial *S. cerevisiae* strain, which showed no difference between conditions, as it was the case for *M. pulcherrima*.

As shown in Table 1, both non-*Saccharomyces* species showed a significant increase in biomass yield when cultivated in diluted molasses in bench-top trials. This increase in biomass yield suggests a more efficient utilization of sugars, which could be explained through a transition to a respiratory metabolism, which is more energetically efficient. A transition to an at least partial respiration of sugars would also be in accordance with the reduction in ethanol production observed for both species in diluted molasses (Table 1). In the case of *S. cerevisiae,* the variations in cellular growth and ethanol production we observed in diluted molasses seem to be a consequence of the reduction in sucrose availability, since there was no variation in biomass yield. Ethanol production in *H. vineae* reflects the higher sugar availability in the case of hydrolyzed molasses and a higher respiratory rate in the case of diluted molasses. In *M. pulcherrima*, the pattern is less obvious, with lower ethanol production in the hydrolyzed and diluted molasses.

### 3.3. Biomass propagation at bioreactor scale

**Figure 6:**
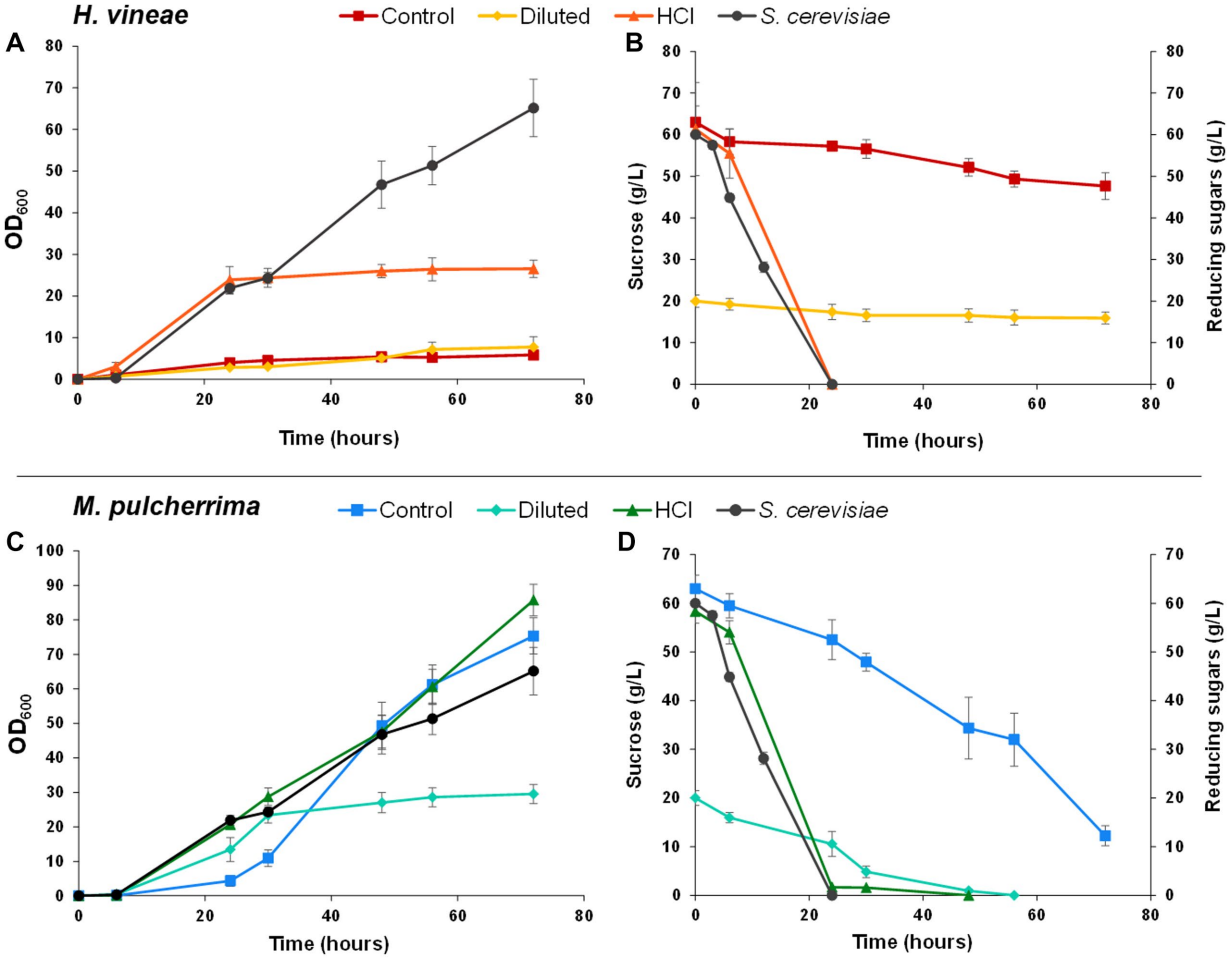
Bioreactor biomass propagation experiments. Panels **(A,C)** show growth curves (OD_600_) of *Hanseniaspora vineae* **(A)** and *Metschnikowia pulcherrima* **(C)** using different culture media: standard molasses (Control), diluted molasses (Diluted) and hydrolyzed molasses (HCl). Panels **(B,D)** show sucrose (g/L) or reducing sugars (g/L) consumption of *H. vineae* **(B)** and *M. pulcherrima* **(D)**. All graphs include *Saccharomyces cerevisiae* data as a reference. Error bars correspond to the SD of three independent experiments.

Bench-top trials are a useful simple tool in understanding the behavior of several yeasts and/or treatments in parallel under industrial propagation conditions. However, in order to work under conditions as close as possible to industrial growth, we performed biomass propagation experiments at a bioreactor scale, which allows a closer control of cultivation conditions. Growth tests for non-*Saccharomyces* species were performed using standard molasses and molasses treated as described previously. *S. cerevisiae* was only grown using standard molasses, since the different treatments did not have any positive effect on cellular growth or biomass yield in bench-top scale experiments (Figures 3, 4). In bioreactor experiments, *S. cerevisiae* showed a standard behavior (Figure 6): initial sucrose was rapidly consumed through a fermentative metabolism during the initial batch phase and was exhausted after approximately 24 h. Once the ethanol produced during the batch phase was consumed, at around 30 h, the fed-batch phase was started. During the fed-batch phase the bioreactor alimentation allowed a respiratory metabolism of sugars which increased biomass yield and cell viability after dehydration (Figure 6; Table 1).

Non-*Saccharomyces* species behavior differs from standard *S. cerevisiae* performance in bioreactor trials. However, *S. cerevisiae* was included in the graphs as a reference. *H. vineae* showed poor growth when sucrose was the main carbon source (control and diluted molasses), as it occurred in bench-top tests, further proving that the inability to metabolize sucrose, due to low invertase activity, seems to be the main challenge *H. vineae* is faced with during standard biomass propagation (Figure 4). When cultivated in hydrolyzed molasses, *H. vineae* showed a growth curve similar to *S. cerevisiae* in the initial batch phase. Furthermore, *H. vineae* catabolized sugars via fermentation, as proven by the high amounts of ethanol produced, which reached their maximum levels at around 30 h (Table 1). Despite the similarities between *H. vineae* and the reference strain in the initial stages of batch growth, we could not perform the transition to the fed-batch phase, as the ethanol produced during the initial phase was not consumed after 72 h, indicating differences in respiration with *S. cerevisiae*.

On the other hand, *M. pulcherrima* showed a very distinct behavior to *S. cerevisiae*. Like *H. vineae*, *M. pulcherrima* was only grown in batch conditions. Unlike in bench-top trials, cellular densities were considerably high at the end of the process and reached in batch conditions OD values similar (control conditions) or even higher (hydrolyzed molasses) than *S. cerevisiae* at the end of the fed-batch phase (Figure 6C). However, there appear to be slight differences among conditions. For instance, when using standard molasses, cellular growth showed an initial lag phase that extended up to 25 h approximately, after which exponential growth began. On the other hand, cells grown in hydrolyzed molasses showed a much shorter lag phase, in accordance with a much faster sugar catabolism (Figures 6C,D). Unlike in the other conditions, cellular growth in diluted molasses was significantly lower, probably due to the lower amount of sucrose available, since sucrose consumption was complete at approximately 48 h, causing a growth arrest. The most notable difference between *M. pulcherrima* and *S. cerevisiae* and *H. vineae* is the strict respiratory metabolism shown by the former in bioreactor tests. When grown in bioreactor, the controlled media aeration allows the respiration of sugars in the Crabtree-negative *M. pulcherrima* (Schnierda et al., 2014), as a consequence, no ethanol production was detected in any growth condition and biomass yield was significantly higher than in bench-top trials (Table 1).

### 3.4. Treatments during biomass propagation do not have detrimental effects on wine quality

As the final use of active dry yeast is wine fermentation, in order to assess the possible effects on wine quality of the different modifications to molasses explored herein, mixed fermentations between *M. pulcherrima* and *S. cerevisiae* were performed. Three co-fermentations were carried out by inoculating active dry yeast of *M. pulcherrima,* previously cultured under different conditions, with active dry yeast of *S. cerevisiae* cultured under control conditions (Figure 5). In all of the experiments the active dry yeast used as inocula were produced at a bioreactor scale. Mixed fermentations with *H. vineae* could not be performed due to the low viability of its dry biomass.

**Figure 7:**
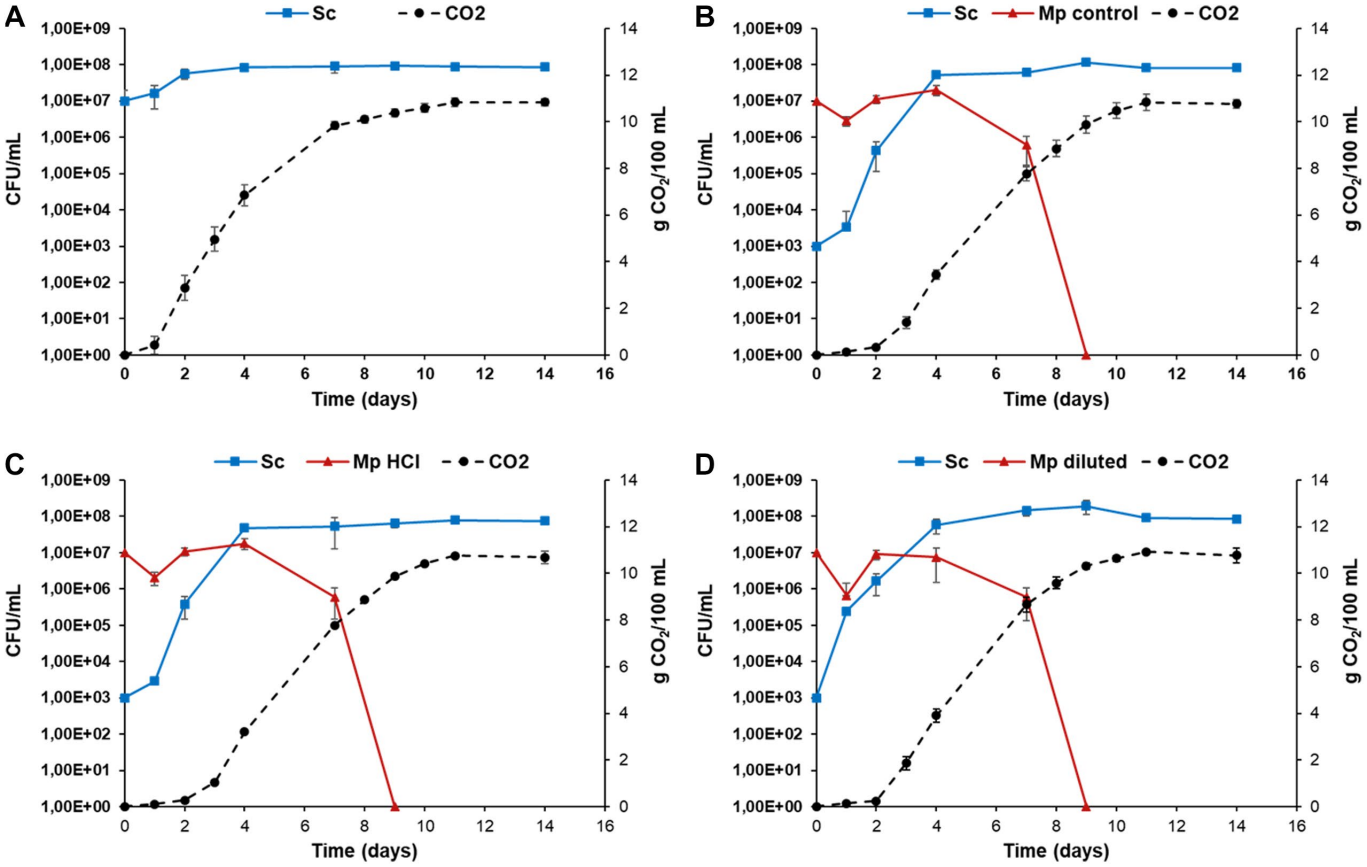
Evolution of yeast population and CO_2_ production during *Metschnikowia pulcherrima* + *Saccharomyces cerevisiae* mixed fermentations and *S. cerevisiae* pure fermentations. Pure *S. cerevisiae* fermentation is shown in panel **(A)**. In mixed fermentations, *M. pulcherrima* previously cultured under different conditions [control molasses **(B)**, hydrolyzed molasses **(C)** and diluted molasses **(D)**] was simultaneously inoculated with *S. cerevisiae.* Error bars correspond to the SD of three independent experiments.

Fermentation kinetics were monitored by measuring CO_2_ levels and cell growth of both species was evaluated during the process (Figure 7). Regarding CO_2_ production, the trend was similar in all of the mixed fermentations. There was no CO_2_ production for the first 2 days of fermentation, after which levels started increasing. On the other hand, pure fermentations with *S. cerevisiae* showed CO_2_ production from the first day. CO_2_ production in mixed fermentations began once *S. cerevisiae* started proliferating. At the end of the fermentative process, the maximum CO_2_ production achieved was around 11 g/100 mL in both the mixed and the pure fermentations. No significant differences were caused by the way *M. pulcherrima* biomass is produced.

**Figure 8:**
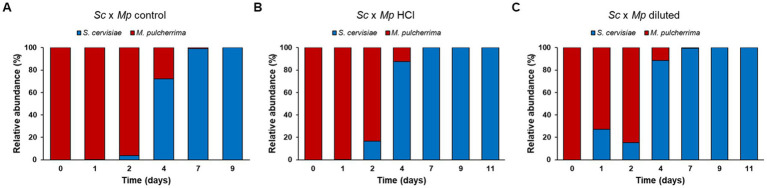
Microbiota evolution in mixed fermentations between *Saccharomyces cerevisiae* and *Metschnikowia pulcherrima* during the first 9 days of fermentation. Bars correspond to the relative abundance (%) of each species in the different mixed fermentations performed.

Likewise, the trend in cell growth in all of the mixed fermentations was similar (Figure 7). *Metschnikowia pulcherrima* exhibited a slight decrease in cell numbers in the first day of fermentation, after which an increase in cell count was observed until the fourth day, at which point *S. cerevisiae* became the predominant yeast in the fermenting grape must. After that, we observed a fast decrease in *M. pulcherrima* population, likely due to the rising ethanol levels (Morata et al., 2019). After 9 days of fermentation, *M. pulcherrima* could not be detected in any fermentation. In all of the mixed fermentations, *S. cerevisiae* cell count increased from the onset of fermentation. *S. cerevisiae* reached its maximum cell counts after the seventh day and remained fully viable until the last day of fermentation (day 14). However, despite the general trend being similar, there are slight differences between fermentations, which become evident when analyzing the relative abundance of each species during the fermentation (Figure 8). In mixed fermentations with *M. pulcherrima* previously cultured in diluted molasses, *S. cerevisiae* was detected already at day 1, whereas in fermentation inoculated with *M. pulcherrima* previously cultured in hydrolyzed biomass *S. cerevisiae* begins to be detected 1 day later. This difference might probably exert an effect on wine composition, as described next.

**Figure 9:**
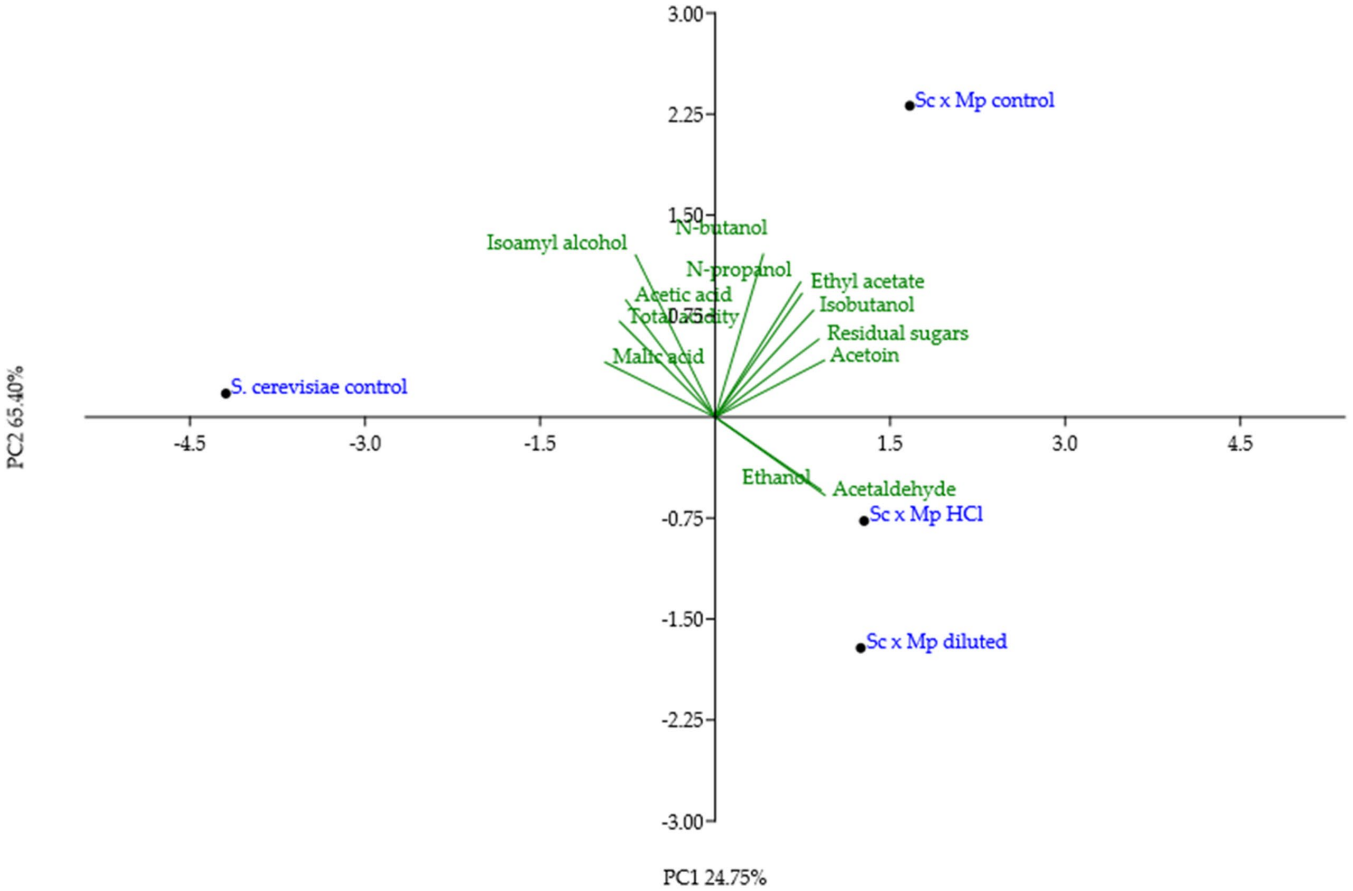
PCA analysis of the data from mixed fermentations between *Saccharomyces cerevisiae* and *Metschnikowia pulcherrima* indicated in Table 2.

Experimental wines obtained in mixed fermentations were analyzed and compared with pure culture wines and among them (Table 2; Figure 9). The presence of *M. pulcherrima* has a clear influence on wine composition, as the PCA analysis show that the control fermentation with just *S. cerevisiae* lies far from mixed fermentations (Figure 9). Ethanol levels and residual sugars did not vary between the pure and mixed fermentations, due to the highly efficient fermentative metabolism and fast propagation of *S. cerevisiae.* pH was slightly lower in mixed fermentations compared to the control fermentation, something already seen in mixed fermentations with *M. pulcherrima* (Escott et al., 2022). Total acidity was comparable among all of the fermentations; in fact, acetic acid production remained similar in all of the fermentations. Nonetheless, we observed slight variations in malic acid content, which was lower in the mixed fermentations than in the pure fermentation. Isobutanol, ethyl acetate, acetoin and to a lesser extent, acetaldehyde levels were statistically higher in mixed fermentations than in pure fermentation, while isoamyl alcohol was the only compound that was found in a lower concentration in mixed wines. Among wines obtained with mixed starters, wine obtained with *M. pulcherrima* growth in control molasses is separated from wines obtained from treated molasses, but this difference is not so high as the second component explains only 25% of the total variance. The only significant differences among them were lower n-propanol and isobutanol levels in mixed fermentations performed with *M. pulcherrima* grown in diluted molasses compared to the other two conditions, probably due to the higher relative abundance of *S. cerevisiae* in this condition. In the case of n-propanol, levels were similar to those of control fermentations. This constitutes one of the most significant results, as it proves that different treatments during biomass propagation did not have a relevant effect (beneficial or detrimental) on wine quality.

**Table 2:** Chemical parameters and volatile compounds detected in wines produced in pure *Saccharomyces cerevisiae* fermentation and in mixed fermentations with *S. cerevisiae* (*Sc*) and *Metschnikowia pulcherrima* (*Mp*) cultured under control conditions, hydrolyzed molasses (HCl), and diluted molasses (diluted).

	*S. cerevisiae* control fermentation	*Sc* × *Mp* control	*Sc* × *Mp* HCl	*Sc* × *Mp* diluted
Ethanol (%)	12.60 (± 0.25)^a^	12.73 (± 0.09)^a^	12.73 (± 0.04)^a^	12.80 (± 0.13)^a^
Residual sugars (g/L)	1.79 (± 0.25)^a^	2.05 (± 0.25)^a^	1.91 (± 0.25)^a^	1.97 (± 0.25)^a^
pH	3.93 (± 0.03)^a^	3.34 (± 0.01)^b^	3.35 (± 0.01)^b^	3.36 (± 0.01)^b^
Total acidity (g/L)	9.63 (± 0.46)^a^	9.51 (± 0.07)^a^	9.36 (± 0.05)^a^	9.44 (± 0.11)^a^
Acetic acid (mg/L)	299.84 (± 46.15)^a^	264.81 (± 10.73)^a^	257.31 (± 9.32)^a^	217.93 (± 37.03)^a^
Malic acid (g/L)	1.87 (± 0.11)^a^	1.63 (± 0.03)^b^	1.52 (± 0.02)^c^	1.59 (± 0.03)^b^
Acetaldehyde (mg/L)	47.95 (± 4.42)^b^	56.98 (± 12.02)^ab^	60.02 (± 8.87)^ab^	61.08 (± 2.21)^a^
N-propanol (mg/L)	16.00 (± 0.88)^c^	23.58 (± 1.02)^a^	21.23 (± 1.76)^ab^	17.47 (± 0.66)^c^
Isobutanol (mg/L)	35.66 (± 1.72)^d^	71.60 (± 4.74)^a^	62.74 (± 7.09)^ab^	48.87 (± 2.39)^c^
N-butanol (mg/L)	17.00 (± 2.42)^ab^	20.26 (± 0.60)^a^	16.42 (± 0.98)^b^	17.89 (± 1.69)^ab^
Isoamyl alcohol (mg/L)	240.85 (± 14.40)^a^	230.84 (± 4.55)^ab^	206.05 (± 15.71)^bc^	198.74 (± 9.27)^c^
Acetoin (mg/L)	4.13 (± 0.35)^b^	6.83 (± 1.09)^a^	5.65 (± 0.54)^a^	6.19 (± 0.02)^a^
Ethyl acetate (mg/L)	19.70 (± 0.80)^c^	29.24 (± 3.83)^a^	27.08 (± 1.77)^a^	21.67 (± 0.59)^b^

All the measurements were taken in triplicate. Different letters denote a significant difference among species and conditions (*p* < 0.05).

## 4. Discussion

Previously published work from our laboratory allowed us to analyze the differences in behavior among various non-*Saccharomyces* species in laboratory scale simulations of active dry yeast production (Torrellas et al., 2020). Several species were found to have diminished growth in molasses when compared to *S. cerevisiae.* Among those studied, we selected *H. vineae* and *M. pulcherrima* due to their oenological interest and the extreme differences shown in dehydration conditions (highly sensitive and tolerant, respectively). In this work, we aim to elucidate the cause for said differences in cellular growth under standard cultivation conditions and to find alternatives to improve the biotechnological performance of these species. The initial hypothesis was that their poor growth in molasses was due to poor sucrose consumption as a result of a limiting ability to properly hydrolyze it, based on what has been described by other authors (Lu et al., 2016; Bellut et al., 2018; Chua et al., 2018). In fact, invertase pretreatment of molasses was required for *M. pulcherrima* to achieve optimal performance (Schnierda et al., 2014). Our data confirms that even after long cultivation times, *H. vineae* and *M. pulcherrima* seem to be unable to fully metabolize the sucrose present in molasses. Moreover, both species showed low invertase in derepressing conditions, a behavior that differs significantly from *S. cerevisiae,* which can hydrolyze sucrose efficiently. Invertase activity has not been previously studied in *H. vineae* and *M. pulcherrima*, so our results would also help to explain their limitation to properly grow on sucrose-based media described by other authors (Lu et al., 2016; Bellut et al., 2018; Chua et al., 2018). Invertase in *S. cerevisiae* is a periplasmic enzyme, so sugars are transported as monosaccharides into the cell. There is no indication that there is such enzyme in the genome of *M. pulcherrima* (Gore-Lloyd et al., 2019). A potential sucrose transporter and intracellular hydrolysis may be an alternative approach, and in fact in the genome of *M. pulcherrima* proteins similar to sucrose transporters and α-glucosidases were found. That alternative pathway may be subject to a regulation that may differ from the described glucose repression of invertase of *S. cerevisiae*. Similarly, invertase *SUC2* gene has been described in *H. vineae* (Carrau et al., 2023), so its expression and regulation may be very different between these two species. It seems that secreted invertase may be not the usual approach to consume sucrose among yeasts.

Sugarcane and beet molasses are the most used media for yeast biomass propagation in industry (Pérez-Torrado et al., 2015). However, despite their widespread use, they appear to be a suboptimal media for the propagation of some non-*Saccharomyces* species. In order to address this issue, we decided to perform simple and easily scalable modifications that could allow a better performance of non-*Saccharomyces* yeasts. These modifications aimed to keep molasses as the basis for biomass propagation, due to its extensive use in industry and its low price. In first place, we opted for the hydrolysis of sucrose. We performed acid hydrolysis with an HCl treatment. Acid hydrolysis has already been employed as a biotechnological strategy to liberate glucose and fructose in molasses for different processes (Quan et al., 2005; Yan et al., 2011). At a bench top scale, molasses acid hydrolysis seemed to be a promising strategy for the improvement of biomass production of both non-*Saccharomyces* species. In fact, in *M. pulcherrima* our results resembled what was previously described in response to enzymatic hydrolysis of sucrose (Schnierda et al., 2014). Furthermore, the treatment with HCl did not have any negative effects on cell viability after dehydration.

As an alternative strategy to molasses hydrolysis, we tested molasses dilution, from 60 g/L sucrose to 20 g/L, in order to test the effect of the sugar concentration reduction on metabolic regulation, plus to dilute any potential inhibitory molecule. This strategy was a particularly interesting approach from a metabolic regulation point of view. When cultivated in diluted molasses cell growth was not improved, but biomass yield was, and viability for *H. vineae* increased greatly suggesting that this approach is better to obtain dry biomass of this sensitive species. Apparently, the reduction in carbon source induced a metabolic shift toward a more efficient respiratory metabolism in both non-*Saccharomyces* species. The mechanisms of metabolic regulation of non-*Saccharomyces* species are yet to be described in depth; hence, we can only speculate with what may be occurring at a molecular level as a response to a reduction in sucrose concentration. In *S. cerevisiae*, the SNF1 pathway promotes alternative carbon source utilization and respiration in response to low glucose levels (Broach, 2012; Conrad et al., 2014). *H. vineae* and *M. pulcherrima* have in their genome sequences with a high identity percentage (74.69% and 73.15% respectively) to the *SNF1* gene of *S. cerevisiae*, suggesting that a similar mechanism may be in play. It is possible that in bench-top trials, the reduction in sugar concentration was enough to trigger a metabolic shift in non-*Saccharomyces* species that favored sugar respiration, which would explain the higher biomass yield and the lower ethanol production we observed. Furthermore, *S. cerevisiae* is a post-WGD (Whole Genome Duplication) yeast, unlike *H. vineae* and *M. pulcherrima* (Wolfe and Shields, 1997; Shen et al., 2016). The increased glycolytic enzyme content in post-WGD yeasts favors sugar fermentation, since the increase in pyruvate concentration promotes its flux to acetaldehyde via pyruvate decarboxylase (Conant and Wolfe, 2007). This, in addition to different transcriptomic regulation of post-WGD yeasts (Ihmels et al., 2005), explains their preference for a fermentative metabolism even in the presence of oxygen and the ability of *S. cerevisiae* to grow at high sugar concentrations, while other yeasts present a slower growth. Understanding the effects of different carbon sources and their concentration on the metabolic flux regulation of non-*Saccharomyces* species may be key in selecting the adequate cultivation conditions to optimize biomass yield at an industrial level.

Bioreactor scale test are needed in order to validate the results obtained at a bench-top scale under conditions closer to industrial wine yeast production. We worked under standard conditions optimized for *S. cerevisiae* production (Pérez-Torrado et al., 2005), with an initial fermentative growth during the batch phase and a transition to a respiratory metabolism during the fed-batch phase, in which biomass yield was increased. The differences between both non-*Saccharomyces* species and the reference strain are accentuated at a bioreactor scale. *H. vineae* only showed an optimal growth when cultivated with hydrolyzed molasses. In an hexose-based media, *H. vineae* presented an initial fermentative growth, with high ethanol production, similar to what has been described for the closely related *H. uvarum* (Escalante et al., 2011). In fact, bioreactor growth in *H. vineae* was similar to *S. cerevisiae* in the initial batch phase. However, the ethanol produced during sugar fermentation was not consumed, so the transition to the fed-batch phase could not be performed. The results obtained at a bioreactor scale confirm that the poor growth shown by *H. vineae* in molasses is due to an inability to properly hydrolyze sucrose. In order to solve this, a media with an hexose-based carbon source is needed for optimal growth. On the other hand, in *M. pulcherrima* the differences between control and hydrolyzed molasses observed in bench-top experiments are minimized at a bioreactor scale. The initial lag phase was shorter in hydrolyzed molasses since hexoses were catabolized at a faster rate than sucrose. Despite that, total cell growth was statistically similar between both conditions at longer cultivation times, due to the optimal respiratory growth at a bioreactor scale. In *M. pulcherrima* the main difference between bench-top and bioreactor trials lies in the effect of the controlled media aeration, which allows sugar consumption via respiration. *Metschnikowia pulcherrima* is a Crabtree negative yeast (Schnierda et al., 2014). In bench-top trials oxygen availability constitutes the limiting factor for a completely respiratory metabolism and sugars are consumed through a less efficient fermentative metabolism. On the other hand, *H. vineae,* like *S. cerevisiae* is a Crabtree positive yeast with a high fermentative capacity (Martin et al., 2018) even when oxygen is available. However, media aeration is still required to allow cells to produce ergosterol (Dimster-Denk and Rine, 1996). This, in addition to the longer cultivation times that allowed a transition to the stationary state and, presumably, the accumulation of reserve carbohydrates, such as trehalose, unlike what occurs in bench-top experiments (Torrellas et al., 2020) may explain the increase in viability after dehydration in bioreactor trials. These results highlight the importance of adapting the biomass propagation process to the requirements of each species. As it occurs in *M. pulcherrima* fed-batch growth may not be needed in other Crabtree negative yeasts of oenological interest such as *Wickerhamomyces anomalus* (Fredlund et al., 2004)*, Kluyveromyces marxianus* (Lane et al., 2011) or *K. lactis* (Siso et al., 1996).

In recent years there has been a reevaluation of the role of non-*Saccharomyces* yeasts in wine, which has allowed to determine their positive contributions to the final product (Ciani et al., 2010; Jolly et al., 2014; Varela, 2016; Morata, 2019). We decided to perform mixed fermentations with active dry yeast of *M. pulcherrima* cultured under different conditions and *S. cerevisiae*, to determine whether the modifications to the biomass propagation process had any effect on wine quality (viable dry yeast from *H. vineae* were not obtained in most conditions). Most laboratory analysis of mixed fermentations are carried out with inocula in stationary phase, but ours were made with dehydrated biomass. Fermentation kinetics differed between both kinds of fermentation. In pure fermentations, *S. cerevisiae* population showed an increase in cell count from the onset of fermentation, while ethanol production started on the first day of fermentation. Due to the presence of *M. pulcherrima* in mixed fermentations, ethanol production was delayed and it only began once *S. cerevisiae* levels started rising. *M. pulcherrima* was the dominant yeast the first 2 days of fermentation but due to its low ethanol tolerance (Morata et al., 2019) it started dying as soon as levels rose. Despite the initial delay in ethanol production, we did not observe an ethanol reduction in mixed fermentations, presumably because the lack of aeration did not allow an efficient sugar respiration by *M. pulcherrima* during the initial stages of fermentation, unlike what has been described by other authors (Quirós et al., 2014; Canonico et al., 2019; Puškaš et al., 2020). There are multiple studies detailing the influence of *M. pulcherrima* on wine secondary compound composition (Ruiz et al., 2018; Morata et al., 2019; Vicente et al., 2020). The influence of *M. pulcherrima* on the composition of the wines produced in this work was evident. In accordance to previously published data, mixed fermentations with *M. pulcherrima* showed an increased content in ethyl acetate (Varela et al., 2016), acetaldehyde (Puškaš et al., 2020), propanol and isobutanol (Comitini et al., 2011; Seguinot et al., 2020), which indicate a clear modification in the aromatic profile of wines, in a direction that should be evaluated by a tasting panel. Nonetheless, one of the most relevant results is that differences in secondary compound composition among the different types of mixed fermentations were minimal. This result proves that the treatments we applied during biomass propagation do not have any detrimental effect on the quality of the final product, proving their applicability for the improvement of the production of non-*Saccharomyces* wine yeasts at an industrial level.

## Data availability statement

The original contributions presented in the study are included in the article/Supplementary material, further inquiries can be directed to the corresponding authors.

## Author contributions

MT designed and performed the experiments and contributed to data interpretation and manuscript writing. RP designed and performed the experiments and contributed to data interpretation. AF-P performed experiments. AC designed experiments, contributed to data interpretation, and revised the manuscript. EM conceived the study, contributed to funding, designed of experiments and data interpretation, and revised the manuscript. AA contributed to funding, designed experiments and data interpretation, revised the manuscript, and approved the final version for publication. All authors contributed to the article and approved the submitted version.

## Funding

Grant PID2021-122370OB-I00 to EM and AA was funded by MCIN/AEI/10.13039/501100011033 and by the European Union FEDER program.

## Conflict of interest

The authors declare that the research was conducted in the absence of any commercial or financial relationships that could be construed as a potential conflict of interest.

## Publisher’s note

All claims expressed in this article are solely those of the authors and do not necessarily represent those of their affiliated organizations, or those of the publisher, the editors and the reviewers. Any product that may be evaluated in this article, or claim that may be made by its manufacturer, is not guaranteed or endorsed by the publisher.

## References

[ref1] Bellut K. Michel M. Zarnkow M. Hutzler M. Jacob F. De Schutter D. P.. (2018). Application of non-Saccharomyces yeasts isolated from kombucha in the production of alcohol-free beer. Fermentation 4:66. doi: 10.3390/fermentation4030066

[ref2] Benito S. Hofmann T. Laier M. Lochbühler B. Schüttler A. Ebert K.. (2015). Effect on quality and composition of Riesling wines fermented by sequential inoculation with non-Saccharomyces and *Saccharomyces cerevisiae*. Eur. Food Res. Technol. 241, 707–717. doi: 10.1007/s00217-015-2497-8

[ref3] Broach J. R. (2012). Nutritional control of growth and development in yeast. Genetics 192, 73–105. doi: 10.1534/genetics.111.135731 22964838 PMC3430547

[ref4] Canonico L. Comitini F. Ciani M. (2019). Metschnikowia pulcherrima selected strain for ethanol reduction in wine: influence of cell immobilization and aeration condition. Foods 8:378. doi: 10.3390/foods8090378, PMID: 31480605 PMC6770742

[ref5] Capece A. Romano P. (2019). “Yeasts and their metabolic impact on wine flavour,” in Yeasts in the production of wine. eds. Romano P. Ciani M. Fleet G. H. (New York: Springer-Verlag New York), 43–80.

[ref6] Capece A. Siesto G. Romaniello R. Lagreca V. M. Pietrafesa R. Calabretti A.. (2013). Assessment of competition in wine fermentation among wild *Saccharomyces cerevisiae* strains isolated from Sangiovese grapes in Tuscany region. LWT Food Sci. Technol. 54, 485–492. doi: 10.1016/j.lwt.2013.07.001

[ref7] Carlson M. Botstein D. (1983). Organization of the SUC gene family in Saccharomyces. Mol. Cell. Biol. 3, 351–359. doi: 10.1128/mcb.3.3.351, PMID: 6843548 PMC368543

[ref8] Carrau F. Dellacassa E. Boido E. Medina K. Valera M. J. Fariña L.. (2023). Biology and physiology of *Hanseniaspora vineae*: metabolic diversity and increase flavour complexity for food fermentation. FEMS Yeast Res. 23:foad010. doi: 10.1093/femsyr/foad010, PMID: 36758966

[ref9] Chua J. Y. Lu Y. Liu S. Q. (2018). Evaluation of five commercial non-Saccharomyces yeasts in fermentation of soy (tofu) whey into an alcoholic beverage. Food Microbiol. 76, 533–542. doi: 10.1016/j.fm.2018.07.016, PMID: 30166185

[ref10] Ciani M. Comitini F. Mannazzu I. Domizio P. (2010). Controlled mixed culture fermentation: a new perspective on the use of non-Saccharomyces yeasts in winemaking. FEMS Yeast Res. 10, 123–133. doi: 10.1111/j.1567-1364.2009.00579.x, PMID: 19807789

[ref11] Comitini F. Gobbi M. Domizio P. Romani C. Lencioni L. Mannazzu I.. (2011). Selected non-Saccharomyces wine yeasts in controlled multistarter fermentations with *Saccharomyces cerevisiae*. Food Microbiol. 28, 873–882. doi: 10.1016/j.fm.2010.12.001, PMID: 21569929

[ref12] Conant G. C. Wolfe K. H. (2007). Increased glycolytic flux as an outcome of whole-genome duplication in yeast. Mol. Syst. Biol. 3:129. doi: 10.1038/msb4100170, PMID: 17667951 PMC1943425

[ref13] Conrad M. Schothorst J. Kankipati H. N. Zeebroeck G. Rubio-texeira M. Thevelein J. M. (2014). Nutrient sensing and signaling in the yeast *Saccharomyces cerevisiae*. FEMS Microbiol. Rev. 38, 254–299. doi: 10.1111/1574-6976.12065, PMID: 24483210 PMC4238866

[ref14] Contreras A. Hidalgo C. Henschke P. A. Chambers P. J. Curtin C. Varela C. (2014). Evaluation of non-Saccharomyces yeasts for the reduction of alcohol content in wine. Appl. Environ. Microbiol. 80, 1670–1678. doi: 10.1128/AEM.03780-13, PMID: 24375129 PMC3957604

[ref15] Dimster-Denk D. Rine J. (1996). Transcriptional regulation of a sterol-biosynthetic enzyme by sterol levels in *Saccharomyces cerevisiae*. Mol. Cell. Biol. 16, 3981–3989. doi: 10.1128/mcb.16.8.3981, PMID: 8754796 PMC231394

[ref16] Dupont S. Rapoport A. Gervais P. Beney L. (2014). Survival kit of *Saccharomyces cerevisiae* for anhydrobiosis. Appl. Microbiol. Biotechnol. 98, 8821–8834. doi: 10.1007/s00253-014-6028-5, PMID: 25172136

[ref17] Escalante W. E. Rychtera M. Melzoch K. Sakoda B. H. Polo E. Q. Cervantes Z. L.. (2011). Actividad fermentativa de Hanseniaspora uvarum y su importancia en la producción de bebidas fermentadas. Rev. la Soc. Venez. Microbiol. 31, 57–63.

[ref18] Escott C. Vaquero C. Loira I. López C. González C. Morata A. (2022). Synergetic effect of Metschnikowia pulcherrima and Lachancea thermotolerans in acidification and aroma compounds in Airén wines. Foods 11:3734. doi: 10.3390/foods11223734, PMID: 36429326 PMC9689907

[ref19] Fleet G. H. (2008). Wine yeasts for the future. FEMS Yeast Res. 8, 979–995. doi: 10.1111/j.1567-1364.2008.00427.x 18793201

[ref20] Fredlund E. Blank L. M. Schnürer J. Sauer U. Passoth V. (2004). Oxygen- and glucose-dependent regulation of central carbon metabolism in Pichia anomala. Appl. Environ. Microbiol. 70, 5905–5911. doi: 10.1128/AEM.70.10.5905-5911.2004, PMID: 15466531 PMC522099

[ref21] Gore-Lloyd D. Sumann I. Brachmann A. O. Schneeberger K. Ortiz-Merino R. A. Moreno-Beltrán M.. (2019). Snf2 controls pulcherriminic acid biosynthesis and antifungal activity of the biocontrol yeast Metschnikowia pulcherrima. Mol. Microbiol. 112, 317–332. doi: 10.1111/mmi.14272, PMID: 31081214 PMC6851878

[ref22] Harkness T. A. Arnason T. G. (2014). A simplified method for measuring secreted Invertase activity in *Saccharomyces cerevisiae*. Biochem. Pharmacol. Open Access 3:1000151. doi: 10.4172/2167-0501.1000151

[ref24] Hu K. Qin Y. Tao Y. S. Zhu X. L. Peng C. T. Ullah N. (2016). Potential of glycosidase from non-Saccharomyces isolates for enhancement of wine aroma. J. Food Sci. 81, M935–M943. doi: 10.1111/1750-3841.13253, PMID: 26954887

[ref25] Ihmels J. Bergmann S. Gerami-Nejad M. Yanai I. McClellan M. Berman J.. (2005). Rewiring of the yeast Transriptional network through the evolution of motif usage. Science 309, 938–940. doi: 10.1126/science.1113833 16081737

[ref26] Jolly N. P. Varela C. Pretorius I. S. (2014). Not your ordinary yeast: non-Saccharomyces yeasts in wine production uncovered. FEMS Yeast Res. 14, 215–237. doi: 10.1111/1567-1364.12111, PMID: 24164726

[ref27] Lambrechts M. Pretorius I. S. (2000). Yeast and its importance to wine aroma. South African J. Enol. Vitic. 21, 97–129. doi: 10.21548/21-1-3560

[ref28] Lane M. M. Burke N. Karreman R. Wolfe K. H. Byrne C. P. Morrissey J. P. (2011). Physiological and metabolic diversity in the yeast Kluyveromyces marxianus. *Antonie van Leeuwenhoek*. Int. J. Gen. Mol. Microbiol. 100, 507–519. doi: 10.1007/s10482-011-9606-x, PMID: 21674230

[ref29] Lleixà J. Martín V. Portillo M. D. C. Carrau F. Beltran G. Mas A. (2016). Comparison of fermentation and wines produced by inoculation of Hanseniaspora vineae and *Saccharomyces cerevisiae*. Front. Microbiol. 7, 1–12. doi: 10.3389/fmicb.2016.00338, PMID: 27014252 PMC4792884

[ref30] López M. C. Mateo J. J. Maicas S. (2015). Screening of β-glucosidase and β-Xylosidase activities in four non-Saccharomyces yeast isolates. J. Food Sci. 80, C1696–C1704. doi: 10.1111/1750-3841.12954 26126488

[ref31] Lu Y. Huang D. Lee P. R. Liu S. Q. (2016). Assessment of volatile and non-volatile compounds in durian wines fermented with four commercial non-Saccharomyces yeasts. J. Sci. Food Agric. 96, 1511–1521. doi: 10.1002/jsfa.7253, PMID: 25966435

[ref32] Martin V. Jose Valera M. Medina K. Boido E. Carrau F. (2018). Oenological impact of the Hanseniaspora/Kloeckera yeast genus on wines — a review. Fermentation 4:76. doi: 10.3390/fermentation4030076

[ref33] Matallana E. Aranda A. (2016). Biotechnological impact of stress response on wine yeast. Lett. Appl. Microbiol. 64, 103–110. doi: 10.1111/lam.12677, PMID: 27714822

[ref34] Morata A. (2019). Enological repercussions of non-saccharomyces species in wine biotechnology. Fermentation 5:72. doi: 10.3390/fermentation5030072

[ref35] Morata A. Loira I. Escott C. del Fresno J. M. Bañuelos M. A. Suárez-Lepe J. A. (2019). Applications of Metschnikowia pulcherrima in wine biotechnology. Fermentation 5:63. doi: 10.3390/fermentation5030063

[ref36] Oro L. Ciani M. Comitini F. (2014). Antimicrobial activity of Metschnikowia pulcherrima on wine yeasts. J. Appl. Microbiol. 116, 1209–1217. doi: 10.1111/jam.12446 24443784

[ref37] Özcan S. Vallier L. G. Flick J. S. Carlson M. Johnston M. (1997). Expression of the SUC2 gene of *Saccharomyces cerevisiae* is induced by low levels of glucose. Yeast 13, 127–137. doi: 10.1002/(sici)1097-0061(199702)13:2<127::aid-yea68>3.0.co;2-# 9046094

[ref38] Pallmann C. L. Brown J. A. Olineka T. L. Cocolin L. Mills D. A. Bisson L. F. (2001). Use of WL medium to profile native flora fermentations. Am. J. Enol. Vitic. 52, 198–203. doi: 10.5344/ajev.2001.52.3.198

[ref39] Pérez-Torrado R. Bruno-Bárcena J. M. Matallana E. (2005). Monitoring stress-related genes during the process of biomass propagation of *Saccharomyces cerevisiae* strains used for wine making. Appl. Environ. Microbiol. 71, 6831–6837. doi: 10.1128/AEM.71.11.6831-6837.2005 16269716 PMC1287652

[ref40] Pérez-Torrado R. Gamero E. Gómez-Pastor R. Garre E. Aranda A. Matallana E. (2015). Yeast biomass, an optimised product with myriad applications in the food industry. Trends Food Sci. Technol. 46, 167–175. doi: 10.1016/j.tifs.2015.10.008

[ref41] Puškaš V. S. Miljić U. D. Djuran J. J. Vučurović V. M. (2020). The aptitude of commercial yeast strains for lowering the ethanol content of wine. Food Sci. Nutr. 8, 1489–1498. doi: 10.1002/fsn3.1433, PMID: 32180958 PMC7063342

[ref42] Quan Z. X. Jin Y. S. Yin C. R. Lee J. J. Lee S. T. (2005). Hydrolyzed molasses as an external carbon source in biological nitrogen removal. Bioresour. Technol. 96, 1690–1695. doi: 10.1016/j.biortech.2004.12.033, PMID: 16023571

[ref43] Querol A. Huerta T. Barrio E. Ramón D. (1992). Dry yeast strain for use in fermentation of Alicante wines: selection and DNA patterns. J. Food Sci. 57, 183–185. doi: 10.1111/j.1365-2621.1992.tb05451.x

[ref44] Quirós M. Rojas V. González R. Morales P. (2014). Selection of non-Saccharomyces yeast strains for reducing alcohol levels in wine by sugar respiration. Int. J. Food Microbiol. 181, 85–91. doi: 10.1016/j.ijfoodmicro.2014.04.024, PMID: 24831930

[ref45] Rapoport A. Golovina E. A. Gervais P. Dupont S. Beney L. (2019). Anhydrobiosis: inside yeast cells. Biotechnol. Adv. 37, 51–67. doi: 10.1016/j.biotechadv.2018.11.003, PMID: 30453013

[ref46] Robyt J. F. Whelan W. J. (1972). Reducing value methods for maltodextrins: chain-length dependance of alkaline 3,5-dinitrosalicylate and chain-length independence of alkaline copper. Anal. Biochem. 45, 510–516. doi: 10.1016/0003-2697(72)90213-8 5060605

[ref47] Roudil L. Russo P. Berbegal C. Albertin W. Spano G. Capozzi V. (2020). Non-Saccharomyces commercial starter cultures: scientific trends, recent patents and innovation in the wine sector. Recent Pat. Food Nutr. Agric. 11, 27–39. doi: 10.2174/2212798410666190131103713, PMID: 30706832

[ref48] Ruiz J. Belda I. Beisert B. Navascués E. Marquina D. Calderón F.. (2018). Analytical impact of Metschnikowia pulcherrima in the volatile profile of Verdejo white wines. Appl. Microbiol. Biotechnol. 102, 8501–8509. doi: 10.1007/s00253-018-9255-3, PMID: 30054701

[ref49] Schnierda T. Bauer F. F. Divol B. van Rensburg E. Görgens J. F. (2014). Optimization of carbon and nitrogen medium components for biomass production using non-Saccharomyces wine yeasts. Lett. Appl. Microbiol. 58, 478–485. doi: 10.1111/lam.12217, PMID: 24447289

[ref50] Seguinot P. Ortiz-Julien A. Camarasa C. (2020). Impact of nutrient availability on the fermentation and production of aroma compounds under sequential inoculation with M. pulcherrima and *S. cerevisiae*. Front. Microbiol. 11, 1–19. doi: 10.3389/fmicb.2020.00305, PMID: 32184771 PMC7058555

[ref51] Shen X. X. Zhou X. Kominek J. Kurtzman C. P. Hittinger C. T. Rokas A. (2016). Reconstructing the backbone of the saccharomycotina yeast phylogeny using genome-scale data. G3 6, 3927–3939. doi: 10.1534/g3.116.034744, PMID: 27672114 PMC5144963

[ref52] Siso M. I. G. Ramil E. Cerdán M. E. Freire-Picos M. A. (1996). Respirofermentative metabolism in Kluyveromyces lactis: ethanol production and the Crabtree effect. Enzyme Microb. Technol. 18, 585–591. doi: 10.1016/0141-0229(95)00151-4 10862875

[ref53] Torrellas M. Rozès N. Aranda A. Matallana E. (2020). Basal catalase activity and high glutathione levels influence the performance of non-Saccharomyces active dry wine yeasts. Food Microbiol. 92:103589. doi: 10.1016/j.fm.2020.103589, PMID: 32950173

[ref54] Tronchoni J. Curiel J. A. Sáenz-Navajas M. P. Morales P. De-la-Fuente-Blanco A. Fernández-Zurbano P.. (2018). Aroma profiling of an aerated fermentation of natural grape must with selected yeast strains at pilot scale. Food Microbiol. 70, 214–223. doi: 10.1016/j.fm.2017.10.008, PMID: 29173630

[ref55] Varela C. (2016). The impact of non-Saccharomyces yeasts in the production of alcoholic beverages. Appl. Microbiol. Biotechnol. 100, 9861–9874. doi: 10.1007/s00253-016-7941-6 27787587

[ref56] Varela C. Sengler F. Solomon M. Curtin C. (2016). Volatile flavour profile of reduced alcohol wines fermented with the non-conventional yeast species Metschnikowia pulcherrima and Saccharomyces uvarum. Food Chem. 209, 57–64. doi: 10.1016/j.foodchem.2016.04.024, PMID: 27173534

[ref57] Viana F. Belloch C. Vallés S. Manzanares P. (2011). Monitoring a mixed starter of Hanseniaspora vineae-*Saccharomyces cerevisiae* in natural must: impact on 2-phenylethyl acetate production. Int. J. Food Microbiol. 151, 235–240. doi: 10.1016/j.ijfoodmicro.2011.09.005, PMID: 21962939

[ref58] Vicente J. Ruiz J. Belda I. Benito-Vázquez I. Marquina D. Calderón F.. (2020). The genus Metschnikowia in enology. Microorganisms 8, 1–19. doi: 10.3390/microorganisms8071038, PMID: 32668690 PMC7409183

[ref59] Wolfe K. H. Shields D. C. (1997). Molecular evidence for an ancient duplication of the entire yeast genome. Nature 387, 708–713. doi: 10.1038/42711, PMID: 9192896

[ref60] Yan D. Lu Y. Chen Y. F. Wu Q. (2011). Waste molasses alone displaces glucose-based medium for microalgal fermentation towards cost-saving biodiesel production. Bioresour. Technol. 102, 6487–6493. doi: 10.1016/j.biortech.2011.03.036, PMID: 21474303

