# Supplementary material for: Optimizing growth and biomass production of non-Saccharomyces wine yeast starters by overcoming sucrose consumption deficiency
Source: Front Microbiol. 2023 Jun 6;14:1209940. doi: 10.3389/fmicb.2023.1209940 (PMC10280074; doi:10.3389/fmicb.2023.1209940)
Supplement: Supplementary file 1 [file Table_1.DOCX]

**Supplemental Table 1.** Sucrose concentration (g/L) in molasses after several treatments with varying HCl concentrations, incubation times and temperatures were tested.
